# Sedentary Behavior Associated with Obesity in Rural-to-Urban Migrant Children by Comparison of Those in Rural and Urban Area in China

**Published:** 2019-11

**Authors:** Jinkui LU, Yongsheng XU, Yongli XU, Gang LIU, Jianming XIANG

**Affiliations:** School of Sport, Shangrao Normal University, Jiangxi Shangrao, P.R. China

## Dear Editor-in-Chief

Obesity is one of the most important and the fastest growing health problem in children all over the world (1). The prevalence of childhood obesity has been rapidly rising in Mainland China also, the 2012 National Survey reported that the prevalence of obesity and overweight was 6.4% and 9.6% among children 6–17 years of age, respectively, having increased by 204.8% and 113.3%, respectively, since 2002 (2). Similarly, it was reported that the prevalence of over-weight/obesity in rural-to-urban migrant children approached that of Shanghai urban children, far exceeding that of rural children (3). A number of studies showed that decreased physical activity and increased television watching are associated with childhood obesity (3–5), as well as the resulting decreased physical fitness (6). In this study, we aimed to examine sedentary behaviors associated with obesity in rural-to-urban migrant children by comparison of those in rural and urban area in China.

**Fig. 1:**
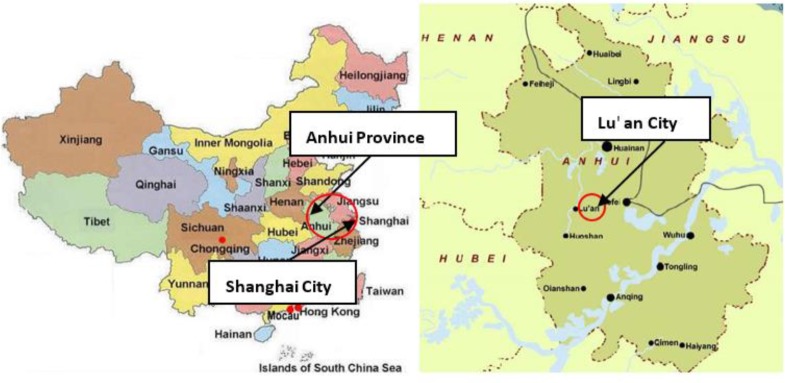
Location of the two areas in China where the study was conducted

This was a cross-sectional study performed from July 2015 to January 2016 in Shanghai and Lu'an rural area, Anhui province, where most of rural to urban migrants came from in this study (Fig. 1).

The final sample included 7–12 years old children 2175 (1111 boys and 1064 girls), 718 in urban Shanghai group, 734 in rural to urban migrants, 723 in rural group.

Informed consent was obtained from the parents of all subjects. The study was approved by the Ethics Committee of School of Physical Education and Health of East China Normal University. Anthropometric measurements and questionnaire investigation was conducted. Continuous variables were expressed as mean ± standard deviation (SD), whereas categorical variables were described as frequencies. Continuous variables were compared using one-way analyses of variance (ANOVA) or independent sample *t*-test, while Chi-square analyses were used to analyze the categorical variables. The associations between obesity and sedentary behaviors were tested multivariable logistic regression models. Odds ratios (ORs) and 95% confidence intervals (CIs) were calculated. All statistical analyses were performed using SPSS 22.0 (IBM, Armonk, NY, USA).

In rural to urban migrant group, the prevalence of obesity was 8.2% in boys, 7.5% in girls; in Urban group, there was 15.4% in boys, 2.1% in girls; in Rural group, boys was 5.4% and girls was 1.7% (Table 1). In the sedentary behaviors’ indications item of “Time of using computer/watching TV every day (h)”, children who use computer/watch TV every day (h) more than 3 hours was 8.6% in Rural group, 25.5% in Rural to urban migrant group, 27.7% in Urban group; Similarly, in the item of “Time of doing homework every day (h), children who do homework more than 3 hour every day was 5.3% in Rural group, 15.7% in Rural to urban migrant group, 21.2% in Urban group.

**Table 1:** Baseline characteristics of the participants by weight status (N=2175)

***Variables ***	***Rural group (n=723)***				***Rural to urban migrant group (n=734)***				***Urban group (n=718)***			
	***Normal***	***Overweight***	***Obesity***	**P**	***Normal***	***Overweight***	***Obesity***	**P**	***Normal***	***Overweight***	***Obesity***	**P**
Gender				<0.001				<0.001				<0.001
Boys, n (%)	279 (74.0)	79(20.8)	19(5.4)		279 (75.6)	59 (16.2)	31(8.2)		231 (61.5)	86 (23.1)	58(15.4)	
Girls, n (%)	280(80.1)	63(18.2)	3(1.7)		285(78.0)	53(14.5)	27(7.5)		272(79.2)	64(18.7)	7(2.1)	
Age (yr)	9.17±0.08	10.08±0.04	10.24±0.07	<0.001	9.61±0.04	9.37±0.05	9.29±0.05	<0.001	9.58±0.06	9.45±0.03	9.73±0.08	<0.001
Body height (cm)	127.17±0.21	130.02±0.38	130.26±0.33	<0.001	132.35±0.30	133.11±0.36	134.57±0.35	<0.001	134.06±0.23	139.59±0.35	138.73±0.38	<0.001
Body weight (kg)	21.28±0.34	23.35±0.46	26.56±0.23	<0.001	30.04±0.37	32.68±0.34	33.85±0.20	<0.001	30.48±0.26	33.63±0.27	34.11±0.38	<0.001
BMI (kg/m2)	14.08±0.05	15.90±0.13	16.13±0.15	<0.001	14.85±0.15	16.21±0.14	17.45±0.15	<0.001	15.91±0.26	16.48±0.31	17.79±0.20	<0.001

Data are shown as mean ± standard deviation or proportions, as appropriate.

BMI: body mass index; Overweight and obesity classification according to the Chinese children and adolescent Standards for BMI

We conducted logistic regressions for both obesity and sedentary behaviors’ indications while controlling for children’ gender and age, in the item of “Time of using computer/watch TV every day (h)”, compared with less than 1 hour every day, rural to urban migrant children who use computer/watch TV more than 3 hours every day were significantly more likely to be obesity (OR=3.56, 95%CI=2.78–4.16). As for the item of “Time of doing homework every day (h)”, compared with less than 1 hour every day, rural to urban migrant children who do homework more than 3 hours every day were significantly more likely to be obesity (OR=3.78, 95%CI=2.90–4.42).

In conclusion, the prevalence of obesity in Rural to urban immigrant children approached that of urban children and far exceeding that of rural children, it attributed to sedentary behaviors, which the migration (Rural to Urban) made lifestyle to be changed.
